# Antisense oligonucleotide targeting Livin induces apoptosis of human bladder cancer cell via a mechanism involving caspase 3

**DOI:** 10.1186/1756-9966-29-63

**Published:** 2010-06-03

**Authors:** Liu Chuan, Wu Xiaohou, Luo Chunli, Hu Zili, Yin Zhikang, He Yunfeng, Du Hu, Zhang Weili, Jiang Qing, Lin Yanjun

**Affiliations:** 1 Department of Urological Surgery, The First Affiliated Hospital, ChongQing Medical University, ChongQing 400016, PR China; 2 Department of Urological Surgery, The Second Affiliated Hospital, ChongQing Medical University, ChongQing 400010, PR China; 3 Faculty of Laboratory Medicine, ChongQing Medical University, ChongQing, 400016, PR China

## Abstract

**Background and Aim:**

in recent years, Livin, a new member of IAPs family, is found to be a key molecule in cancers. Researchers consider Livin may become a new target for tumor therapy; however, the role of it in bladder cancer is still unclear. The purpose of this article is to investigate Antisense Oligonucleotide (ASODN) of Livin on treating bladder cancer cell and underlying mechanisms.

**Methods:**

Phosphorathioate modifying was used to synthesize antisense oligonucleotides targeting Livin, followed by transfection into human bladder cancer cell 5637. After transfection, Livin mRNA and protein level, cell proliferation and apoptosis changes, caspase3 level and its effect on human bladder cancer transplantable tumor in nude mice were measured.

**Result:**

results showed Livin ASODN effectively inhibited Livin expression and tumor cell proliferation, and these effects probably through enhanced caspase3 activity and apoptosis of tumor cells. In nude mice transplantable tumor model, Livin expressions were inhibited meanwhile caspase3 expression was increased. Tumor growth slowed down and apoptosis was enhanced.

**Conclusion:**

Our data suggest that Livin plays an important role in inhibiting apoptosis of bladder cancer cells. Livin ASODN may promote cell apoptosis, inhibit bladder cancer growth, and become one of the methods of gene therapy for bladder cancer.

## Introduction

Inhibition of apoptosis is one of the important mechanisms for the growth of many malignant tumor cells. IAPs, the new anti-apoptotic protein families which independent of Bcl-2, are a hot apoptosis research field in recent years, and can play an important role in inhibiting tumor cell growth. Until now, 8 members of IAPs family were found: NAIP[1], ILP-2[2], c-IAPl(MIHB, HIAP-2), c-IAP2((HIAP-1, MIHC, API2)[3], XIAP(hILP, MIHA, ILP-1)[4], Bruce(apollon)[5], survivin[6] and Livin(ML-IAP, KIAP)[7]. Livin as a new member of IAPs family was found in recent years, which shows high expression level in some specific tumor tissue cells, but little, if not none, in normal tissues. Researchers had found that it may become the target for tumor therapy [8,9]. In 2003, Gazzaniga et al [10] used RT-PCR in 30 cases of transitional cell carcinoma of the bladder (TCCB) tumor tissue to detect Livin mRNA expression level, and the results showed that normal bladder tissues did not express Livin, while TCCB tissues expressed high level of Livin. They made a follow-up visit for 4 years to these patients and finally discovered that the Livin positive expression was quite related to the tumor recrudescence. So the objective of this study is to apply antisense oligonucleotide for Livin gene to investigate the effect of inhibition Livin expression on proliferation and apoptosis of human bladder cancer cell 5637 in vivo and in vitro, and to further explore the mechanisms under the phenomenon, and to provide a theoretical basis for treatment of bladder cancer using antisense oligonucleotide with Livin as a target gene.

## Materials and methods

### Synthesis of antisense oligonucleotide

Livin antisense oligonucleotide sequence was from the literature [11], and a misantisense oligonucleotides (MSODN) was also designed. According to Genbank, ASODN and MSODN do not match with any known mammalian gene. They were synthesized by Takara Biotechnology Co., Ltd (Dalian, China) with phosphorathioate oligonucleotide technology followed by PAGE purification. Using serum-free and antibiotic-free RPMI1640 medium to dilute the stock solution to 20 μmo1/L followed by filtration of microporous filtering film and preservation at -20°C. Antisense sequence: 5'-ACCATCACCGGCTGCCCAGT-3', target sequence: 5'-ACUGGGCAGCCGGUGAUGGU-3', missense sequence: 5'-GTCAGGATCTTCCCACGGAG-3'.

### Culture and transfection of human bladder cancer cell line 5637

Human TCCB cell line 5637 was purchased from the Institute of Cell Research, Shanghai, and Chinese Academy of Sciences. The cells were cultured in RPMI 1640 medium (Gibico, U.S.A.) supplemented with 10% fetal bovine serum (FBS, Sijixin Inc., China) and 1% penicillin-streptomycin (Invitrogen, U.S.A.). All cells were cultured in 6-well plate at 37°C with 5% CO2. During the logarithmic growth phase, the liposome was respectively mixed with antisense and missense oligonucleotides in serum-free medium (Invitrogen, USA) in accordance with Lipofectamine™ 2000 (Invitrogen, USA) instructions to form liposome-oligonucleotide complexes, which were then added into culture plate. The final concentration of oligonucleotide was 160 nmolL^-1^. Seventy-two hours after transfection, cells were harvested for RT-PCR, Western Blot, cell immunofluorescence, flow cytometry analysis, transmission electron microscope observation and Caspase3 activity measurement.

### 3-(4, 5-Dimethylthiazol-2-yl)-2, 5-dimethyl tetrazolium bromide (MTT) assay for cell inhibition

Cells in logarithmic growth phase were seeded in 96-well plates at 5 × 10^4 ^cells per well. Then cells were transfected with antisense oligonucleotide of different concentrations (the final concentrations are 0 nmol/L, 20 nmol/L, 40 nmol/L, 60 nmol/L, 80 nmol/L, 100 nmol/L, 120 nmol/L, 140 nmol/L, 160 nmol/L, 180 nmol/L, 200 nmol/L) for 6 hr, followed by culturing with nomal medium for 66 hr. Four hours before stop culturing, 20 μL of 5 mg/mL MTT (sigma, U.S.A.) was added to the culture medium. After incubation, the culture medium was removed and 200 μL of dimethylsulphoxide(DMSO) was added to resolve the crystal. Absorbance was measured at 490 nm. Each sample was assayed for four times. Tumor cell inhibition rate = (1 - treated group absorbance/control group absorbance) × 100%.

### Semiquantitative RT-PCR

Total RNA was extracted from tissue homogenates or cell lysates with TRIzol reagent (invitrogen, U.S.A.) and RT-PCR was carried out with a RNA PCR Kit Ver.3.0 (TaKaRa, Japan) according to the kit's instructions. Livin-specific primers discriminating between the α- and the β-variant were: forward, 5'-GTCCCTGCCTCTGGGTAC-3'; reverse, 5'-CAGGGAGCCCACTCTGCA-3'. Product sizes 368 and 314 bp, respectively. The primers used for GAPDH were: forward, Sense: 5'-ATGACATCAAGAAGGTGGTG-3'; reverse, 5'-CATACCAGGAAATGAGCTTG-3', which yields a product of 177 bp. The PCR condition was: 95°C for 2 min, then 38 cycles at 94°C for 30 seconds, 64°C for 45 seconds, and 72°C for 30 seconds in 1.5 mM MgCl2-containing reaction buffer. Five μL of RT-PCR products were resolved on 1.5% agarose gels. The gels were stained with ethidium bromide (EB) and were scanned for densitometric estimation of the Livin products with GAPDH products serving as the internal control.

### Western Blot Analysis

Proteins were extracted from cell lines as previous described: Proteins were separated by 12% SDS polyacrylamide gel electrophoresis, transferred to an nitrocellulose membrane (Millipore, USA), and detected with a monoclonal anti-β-actin antibody (R&D systems, USA) or polyclonal anti-Livin antibody (R&D systems, USA), employing enhanced chemiluminescence (PIERCE, USA).

### Immunofluorescence, immunohistochemical and laser confocal microscope for expression of Livin

Cells were transfected with different reagents 72 hours and then disposed the culture medium. Use 0.01 mol/L PBS to rinse, and 4% paraformaldehyde to fix at room temperature followed by reaction with 0.4% Triton-X100 at room temperature for 20 min. Add rabbit serum followed by reaction for 30 min at room temperature. Add primary antibody (goat anti-human Livin, R&D systems, USA) and place it in a wet box for overnight at 4°C followed by 0.01 mol/L PBS rinse. Add secondary antibody labeled with FITC (rabbit anti-goat) and set it in a wet box at room temperature for 1 h followed by 0.01 mol/L PBS rinse and 50% glycerol mounting. Use laser scanning confocal microscope (Leica Tcs Sp2) for observation and imaging.

For immunohistochemical examination, tumor tissue samples were fixed with 4% paraformaldehyde for 72 hr, dehydrated in graded ethanol, and embedded in paraffin followed by serial sections. SP kit, goat anti-human Livin antibody, rabbit anti-human Caspase3 antibodies were purchased from the American R&D systems. Immunohistochemistry staining: repair the antigen with trypsin, add goat anti-human Livin antibodies or rabbit anti-human Caspase3 antibody followed by PBS washing, DAB color development

### Transmission electron microscope for cell morphology

After transfection, the cells in each group were centrifuged at 1500 rpm for 20 min, followed by fixing with 4% paraformaldehyde for 1 hr, and then transferring into pre-chilled 1% glutaraldehyde. The samples were dehydrated in graded ethanol, embedded in epon 812, and then cut into ultrathin or semithin sections. The sections were stained and examined under a Hitachi H-600 transmission electron microscope.

### Detection of apoptotic cells by flowcytometry

Cells were collected by low speed centrifugation and washed with ice-cold PBS then recollected by centrifugation. After washing with PBS twice the cells were incubated in 10 μl Annexin V-FITC (fluorescein isothiocyanate) and 5 μl propidium iodine (PI) at 4°C for 30 minutes using the Annexin V-FITC apoptosis assay kit (KeyGen Biotech. Co. Ltd. Nanjing, China). Finally, the cells were analyzed within 60 minutes by flow cytometry.

### Determination of Caspase3 activities by kinase assay

The experiments conformed to the operating instructions provided by the kit (BioVision Inc.): cells were collected and added with cell lysis solution followed by incubation on ice for 10 min and centrifugation. The supernatant was added with reaction buffer and coupling substrate followed by 37°C water bath for 1 h. The absorbance values were determined by enzyme-linked assay at 405 nm wavelength. The values were regarded as the relative activity of Caspase3.

### Nude mouse xenograft model

Female BALB/c nude mice, 4 weeks of age, weighting 16 ± 0.6 g, were purchased from Experimental Animal Center of Chinese Academy of Sciences, Shanghai, China. Mice were housed in microisolator cages in a specific pathogen-free (SPF) condition with 12-hr light-dark cycles. Mice were subcutaneously implanted with 1 × 10^7 ^5637 cells. Once tumors reached approximately 60 μL in volume, the mice were allocated to receive either ASODN or MSODN treatment, with the concentration of 200 nmol/L and 0.2 ml/mice. The nude mice injected with ASODN were termed as treatment group and the nude mice injected with MSODN were termed as control group. Complexes of ASODN or MSODN plus 4 μL invivo-jetPEI™ (polyplus-transfection Inc., U.S.A.) and also plus 160 μL 5% glucose were directly injected into the tumor once every other day with a total of 7 times. Tumor dimensions were measured once every three days and the tumor volumes calculated using the formula: 1/2 × a × b^2^, where a and b respectively represented the larger and smaller tumor diameter. At the end of the treatment, mice were killed by overdose of ketamine (400 mg/kg) and xylazine (50 mg/kg) and necropsy was performed. Tumor tissue samples were prepared for Immunohistochemistry or TUNEL cell apoptosis detection. Tumor growth inhibition (TGI) was calculated using the formula TGI (%) = (1-M_T_/M_C_) × 100, where M_T _and M_C _are the mean tumor masses in the treatment group and control group respectively.

### TUNEL analyses for cell apoptosis detection

For detection of apoptosis, TUNEL analyses were performed using the in situ cell death detection kit (Roche Molecular Biochemicals, USA). Operations were carried out according to kit instructions. 10 high-powerfields were selected for each case. Count the number of apoptotic cells and total number of cells for each powerfield to calculate the percentage of apoptotic cells (number of apoptotic cells in each powerfield/total cell number in each powerfield) i.e., apoptosis index (AI).

### Statistical analysis

The results were expressed as mean ± standard deviation. One-way analysis of variance (ANOVA) was used to determine the levels of difference between all groups. Comparisons for all pairs were made using Student-Newman-Keuls (SNK) test. p < 0.05 was considered statistically significant.

## Results

### Livin antisense oligonucleotide dose-dependently inhibit bladder cancer cell growth

**Figure 1:**
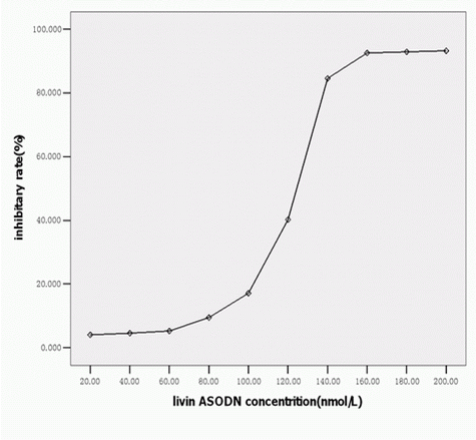
**Inhibitory rate of 5637 cells transfected with Livin ASODN**. After the transfection of bladder cancer cell lines with different concentrations of Livin antisense oligonucleotides, the inhibition effect on cell growth was determined by MTT and a clear dose-dependence was found.

After transfected with different concentrations of Livin antisense oligonucleotides, cell growth of bladder cancer cell lines was determined by MTT and an obvious dose-dependently inhibitory effect was found (Fig 1). When the Livin antisense oligonucleotide concentration was 160 nmol/L, the cell growth inhibition rate reached 92.61 percent, although reagent concentration was continuously increasing, the inhibition rate will not increase significantly (P > 0.05). Accordingly, we chose 160 nmol/L oligonucleotide as the suitable concentration for further study.

### Livin mRNA and protein expression was inhibited after Livin ASODN transfection

**Figure 2:**
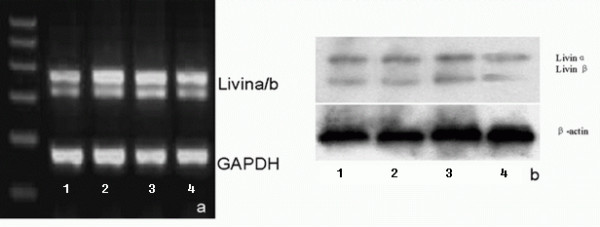
**Livin mRNA and protein expression level in each group of cells**. After transfected with Livin antisense oligonucleotides, (a) the Livin mRNA was decrease significantly (Lane 1: antisense group; 2: missense group; 3: Lipo group; 4: PBS group) and (b) the Livin protein was decrease significantly(Lane 1: PBS group; 2: missense group; 3: Lipo group; 4: antisense group;), while the other three groups did not have significant difference.

To demonstrate the inhibitory effect of Livin ASODN on Livin expression, RT-PCR, Western blot, and LSCM were applied to detect the Livin mRNA and protein expression level in the cells of each group. In RT-PCR experiment, Livin gene electrophoretic bands were seen at the positions of 314 bp and 368 bp relative to Marker in each group, which demonstrated that 5637 cells expressed Livinα and Livinβ. However, the brightness of the electrophoretic bands in antisense group was significantly lower than the one in missense group, liposome group and PBS group; while the brightness of the last three groups were similar (Fig. 2a).

Then we performed Western blot to evaluate Livin protein expression. Confirmed with the results of RT-PCR, the expression of Livinα and Livinβ in the antisense group was significantly lower than the ones in missense group, liposome group and PBS group, while the expression of Livinα and Livinβ in the last three groups were similar (Fig. 2b).

**Figure 3:**
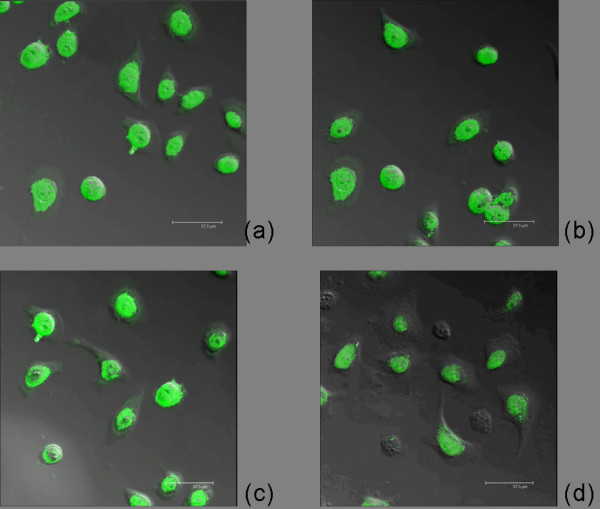
**Using confocal laser scanning microscope detects Livin Expression and location**. After the transfection of Livin ASODN, the green fluorescence for marking Livin significantly decreased with uneven distribution. It was observed that the volume of some cells significantly reduced with no green fluorescence at all, while the other three groups did not have significant difference. (a: PBS group; b: Lipo group; c: missense group; d: antisense group).

Using laser scanning confocal microscopy (LSCM) images, we found Livin-*ir *located in the cytoplasm and nucleus with the majority in the nucleus. The intensity and distribution of Livin-*ir *in PBS group, liposome group and missense group cells were similar. After the transfection of Livin ASODN, the green fluorescence for marking Livin significantly decreased with asymmetrical distribution. It was observed that the volume of some cells even significantly reduced with no green fluorescence at all (Fig. 3). Together, these data demonstrated that Livin mRNA and protein expression were inhibited after Livin ASODN transfection.

### Cell morphology changed and apoptosis rate increased after transfection with Livin ASODN

**Figure 4:**
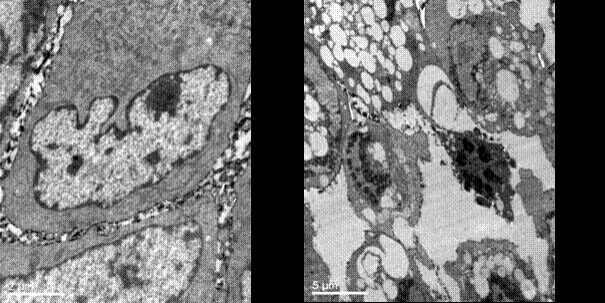
**The morphologic changes of each group cells observed by electromicroscope**. Antisense group showed more cell degeneration and necrosis, with cell volume enlargement, chromatin margination and dissolving and lipid droplets within the cytoplasm increase, endoplasmic reticulum dilation, and swelling of mitochondria like vacuoles. Occasional plasma membrane rupture and cell collapse were also seen. (a: control group(original magnification × 10000 b: antisense group(original magnification × 4000).

As transfection Livin ASODN can inhibit bladder cancer cell growth, we next wanted to confirm the mechanisms underlying this inhibitory effect. Using transmission electron microscopy we found: Antisense group showed more cell degeneration and necrosis, with cell volume enlargement, chromatin margination and dissolving and lipid droplets within the cytoplasm increase, endoplasmic reticulum dilation, and swelling of mitochondria like vacuoles were observed. Occasional plasma membrane rupture and cell collapse were seen. And a small amount of apoptotic cells could also be seen: cell volume reduced, matrix electron density increased, nuclear membrane invaginated, chromatin agglutinate until broken into many small pieces. Cell plasma membrane inward shrunk with the formation of apoptotic bodies in which nuclear materials were visible. No significant changes in cell morphology occurred in the other three control groups (Fig. 4).

**Figure 5:**
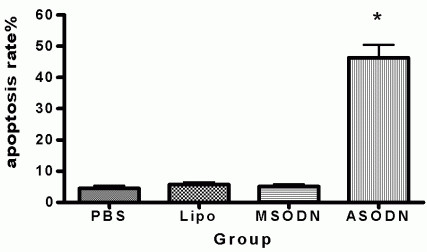
**Cell apoptosis rate measurement**. Antisense group showed increase of cell apoptosis rate (46.39 ± 9.23) %, while the other three groups did not have significant difference. *, p < 0.05.

To further confirm the increasing apoptosis rate, we used flow cytometry to measure cell apoptosis. The results showed cell apoptosis rate of antisense group with Livin ASODN transfection (46.39 ± 9.23) % was significantly higher than PBS group (4.54 ± 1.84) %, liposome group (5.70 ± 1.61)%, and missense groups (5.10 ± 1.56)% with P < 0.01. The apoptosis rates of the latter three groups had no significant difference, P > 0.05. (Fig. 5).

### Cellular caspase3 activities were increased after transfection with Livin ASODN

**Figure 6:**
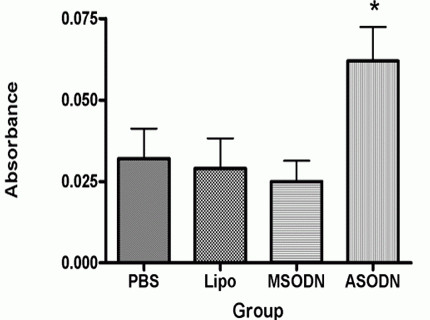
**Caspase3 activities in the cells of each group**. The results of kinase method to detect Caspase3 activity showed that after Livin ASODN transfection with 5637 cells, the Caspase3 activity was significantly increased with the relative activity of 0.062 ± 0.018. Compared with missense group (0.025 ± 0.011), liposome group (0.029 ± 0.016) and PBS group (0.032 ± 0.016), the difference was significant with P < 0.05. The latter three groups had no significant difference *, p < 0.05.

As Caspase3 is an important apoptosis inducing kinase, we next detect the Caspase 3 activity in bladder cancer cells after transfect with Livin ASODN. Results of Caspase3 activity kinase method showed that after Livin ASODN transfection into 5637 cells, the Caspase3 activity was significantly increased with the relative activity of 0.062 ± 0.018 (fig 6). Compared with missense group (0.025 ± 0.011), liposome group (0.029 ± 0.016) and PBS group (0.032 ± 0.016), the difference was significant with P < 0.05. The latter three groups had no significant difference, P > 0.05. all this result indicated that Livin ASODN may through increasing Caspase 3 activity to induce bladder cancer cell apoptosis and thus inhibit its growth.

### Livin ASODN transfection inhibited 5637 tumor growth in vivo

**Figure 7:**
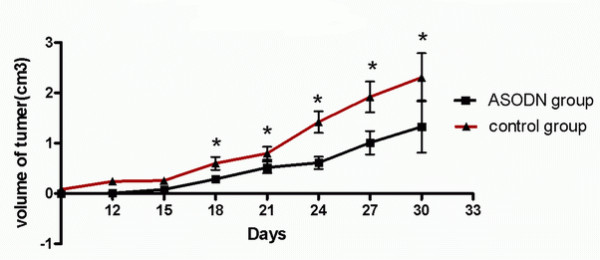
**Comparison of tumor volume in nude mice injected with MSODN and ASODN**. After injection of Livin ASODN, tumor volume was significantly smaller in ASODN group than in MSODN group from 18 to 30 days. Tumor growth was inhibited by injection of ASODN compared with injection of MSODN. *, p < 0.05.

As we had confirmed that Livin ASODN can effectively inhibit bladder cancer cell growth by increasing its apoptosis, next we want to know whether this treatment effect will appear in in vivo experiments. Nude mouse xenograft model was describe as materials and methods previously, and tumor growth was observed continuously. In addition, tumor size was measured and calculated at different times, and draw tumor growth curves. The results showed that compared with the control group, the tumor volume of antisense was significantly smaller than the one of control group, P < 0.05 (Fig. 7) from the 18th day after tumor inoculation until the 30th day, which indicated that the injection of Livin ASODN inhibited tumor growth. 30 days after inoculation of 5637 cells, the tumor weight of MSODN injection group was 2.41 ± 0.41 g and the tumor weight of ASODN inoculation group was1.31 ± 0.88 g. t tests showed that the tumor weight of two groups had significant difference with P < 0.05.

### Cell apoptosis was induced after transfected with Livin ASODN in vivo

**Figure 8:**
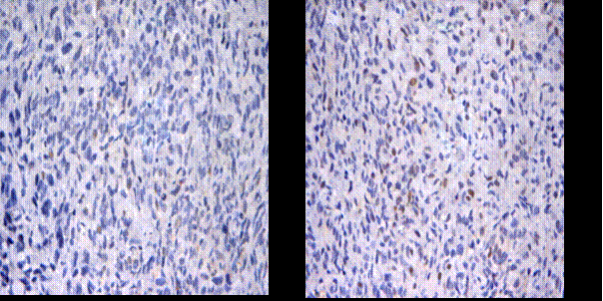
**Apoptosis in tumor tissue of nude mice observed by TUNEL staining**. The tumor cell nucleus of Livin ASODN injection group was stained brown-red with nuclear enrichment. And the cytoplasm was dispersedly and slightly stained. Randomly select 10 high power fields for each case to calculate the apoptotic index (AI). The antisense group apoptotic index was 19.60 ± 5.91, which was significantly higher than the control group (3.48 ± 2.35), and the difference was significant with P < 0.05(a Control group, b Livin ASODN group) (original magnification ×400).

The microscope observation after TUNEL staining showed that the center of positive cell nucleus was round and uniform brown, which was the sign of DNA fragmentation after TUNEL reaction in cells. The negative cells had no cell morphological changes and were not colored or only slightly stained. The results showed that: the tumor cell morphologic of MSODN injection group was normal. Only a small amount of cell nucleus was colored and the cytoplasm was slightly stained. However, the tumor cell nucleus of Livin ASODN injection group was stained brown-red with nuclear enrichment. And the cytoplasm was dispersedly and slightly stained (Fig. 8).

Randomly select 10 high power fields for each case to calculate the apoptotic index (AI). The antisense group apoptotic index was 19.60 ± 5.91, which was significantly higher than the control group (3.48 ± 2.35), and the difference was significant with P < 0.05.

### Livin expression was inhibited meanwhile Caspas 3 expression was increased after transfection with Livin ASODN

**Figure 9:**
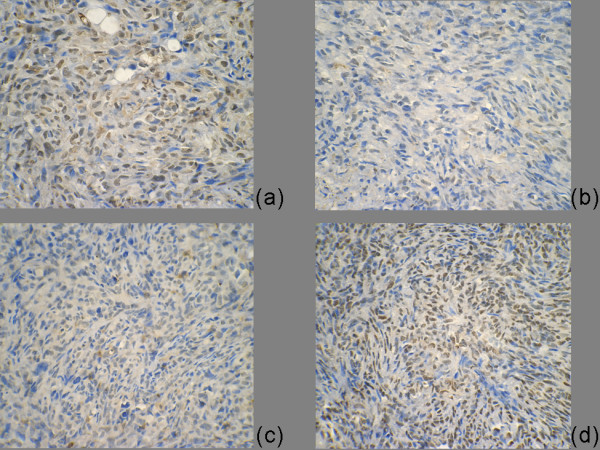
**Livin and caspase 3 expression level in tumor tissue of nude mice**. After injection of Livin ASODN, the Livin expression level in tumor tissue was significantly inhibited (a control group compare b ASOND group) and Caspase 3 expression was significantly increased (c control group compare with d ASOND group).

Livin immunohistochemistry showed that the majority of tumor cells in the tumor tissues of MSODN injection group were stained dark brown, while the tumor cell nucleus of ASODN injection group was stained pale yellow and the number of stained cells was small (Fig. 9a, b).

Caspas 3 immunohistochemical staining showed that the majority of tumor cells in the tumor tissues of ASODN injection group were stained dark brown, while the tumor cell caspas 3 staining of MSODN injection group was relatively light, and the number of stained cells was relatively small (Fig. 9c, d).

## Discussion

In recent years, using EST clone containing the BIR sequences, Vucic D, Kasof GM, etc. found Livin--a new member of this gene family in human fetal kidney cDNA library according to the IAPs homologous sequences [12,13]. Livin produces two kinds of mRNA isomers in the transcription process due to the different ways of splicing. They have 1351 and 1297 base pairs, respectively. In spite of the 54 bp difference in length, properties of these two different mRNAs are exactly the same. The proteins coded by them were 298 and 280 amino acids with the molecular weight of about 33 kD and 30 kD, and were respectively termed as Livin α and Livin β [14]. For normal adults, most tissues do not express Livin at all (except placenta), but in some cancer cell lines such as melanoma cell lines (G361 and SK-Mel29), lymphoma, HaCat cells, and MCF7 breast cancer cells [14], Livin is highly expressed. In spite of that, Livin was also highly expressed in a number of tumor tissues, such as bladder cancer [10], lymphoma [13], lung cancer [15], hepatocellular carcinoma[16], and renal carcinoma[17,18]. Gazzaniga et al screened the apoptosis-related genes in bladder transitional cell carcinoma tumor tissues, including Livin, Survivin, BCL-X and BCL-2/BAX and so on, and then performed a four-year follow-up visit. Results showed that only Livin was related to bladder cancer recurrence in these genes[10]. The tumor average recurrence time of the patients with positive Livin expression after surgery (3.5 months) was much less than the one of the patients with negative Livin expression (27.2 months). The significant differences of recurrence intervals indicated that Livin expression is a sign of poor prognosis of early superficial bladder cancer and it can be used as indicators for monitoring recurrence of bladder cancer. As one of the most common malignant tumors in urinary surgery, the bladder cancer has the most remarkable characteristics of easily postoperative recurrence. Therefore, Livin as a target gene for treating bladder cancer has a good application prospect.

Antisense nucleic acid is a naturally existing or synthetic nucleotide sequence. Livin ASODN hybridizes with target genes through Watson Crick principle of complementary base pairing to prevent gene expression, inhibit cell proliferation, promote apoptosis, and achieve the purpose of preventing or treating tumors. The natural oligonucleotide is easily degraded, but phosphorathioate modifying can increase the capacity of its tolerance to nucleic acid hydrolysis, with good solubility and hybridization properties. The effectiveness and safety have been universally accepted by researchers. Currently the antisense oligonucleotide with bcl-2 as the target gene (trade name: Oblimersen) is in Phase III clinical trials with the permit of FDA (mainly treat malignant melanoma, chronic lymphocytic leukemia, multiple myeloma, etc.) [19]. The drug achieves the purpose of cancer treatment by inhibiting the expression of bcl-2 inside the tumor cells and inducing the tumor cell apoptosis. There are also a variety of antisense oligonucleotides anticancer drugs in clinical trials [20,21]. In the present study, phosphorathioate modifying greatly enhanced the anti-ribozyme decomposition capacity of Livin ASODN. The supplement of cationic liposome transfection further increased its stability and improved the ability of uptake by cells. Using RT-PCR, Western blot, immunocytochemistry, immunohistochemistry, we found that Livin ASODN could inhibit the expression of Livin mRNA and protein. We further observed that the cell growth was inhibited and the apoptosis increased from MTT, flow cytometry, TUNEL method and morphological observations.

Caspases protein plays an important role in apoptosis. Most of the stimuli induce apoptosis through the Caspase protein cascade activation reactions. Caspases protein family has more than 10 members. Literatures have reported that Livin can interact with Caspase-3, -6, -7, -8, -9, -10 [22] (especially Caspase 3) to inhibit the process of apoptosis. Using immunohistochemistry, we observed that after the injection of Livin ASODN, the expression of Caspase 3 in tumor tissues increased, which was probably because Livin ASODN inhibited the expression of Livin and then removed the binding inhibition to Caspase 3. Besides, Caspase 3 removal function also enhanced, which lead to increased cell apoptosis.

**In conclusion**, Livin ASODN could specifically inhibit the expression of Livin in human bladder cancer cell 5637 and induce apoptosis of bladder cancer cells. It may be a potential and most promising strategy for bladder cancer.

## Competing interests

The authors declare that they have no competing interests.

## Authors' contributions

LC is the first author of this paper and the most designing work was done by him; WXH is the corresponding author. LCL carried out the cells experiments; HZL carried out the transmission electron microscopy observation;YZK carried out the immunohistochemical staining;HYF and DH participated in the study design;ZWL carried out the data collection and statistic work; JQ and LYJ carried out the animal works. All authors read and approved the final manuscript
